# Ovulation stigma and rupture of ovarian tunica albuginea in a patient with polycystic ovary syndrome (video)

**DOI:** 10.1093/jscr/rjab482

**Published:** 2021-10-31

**Authors:** Nawras Alhalabi, Mohammad Marwan Alhalabi, Sawsan Alsamawi, Marwan Alhalabi

**Affiliations:** Faculty of Medicine, Damascus University, Damascus, Syria; Faculty of Medicine, Damascus University, Damascus, Syria; Assisted Reproduction Unit, Orient Hospital, Damascus, Syria; Assisted Reproduction Unit, Orient Hospital, Damascus, Syria; Division of Reproductive Medicine, Embryology and Genetics, Faculty of Medicine, Damascus University, Damascus, Syria

## Abstract

A 25-year-old patient was referred to the fertility clinic in our hospital, complaining of primary infertility for 3 years. The patient reported having irregular menses, and physical examination showed clinical hirsutism, while transvaginal ultrasound revealed polycystic ovarian morphology. Hormonal workup showed elevated Anti-Müllerian Hormone (7 ng/ml), and normal levels of prolactin and thyroid stimulating hormone. Hysterosalpingogram revealed bilateral obstruction of fallopian tubes. Semen analysis of the husband was within normal limits.

## INTRODUCTION

**Figure 1:**
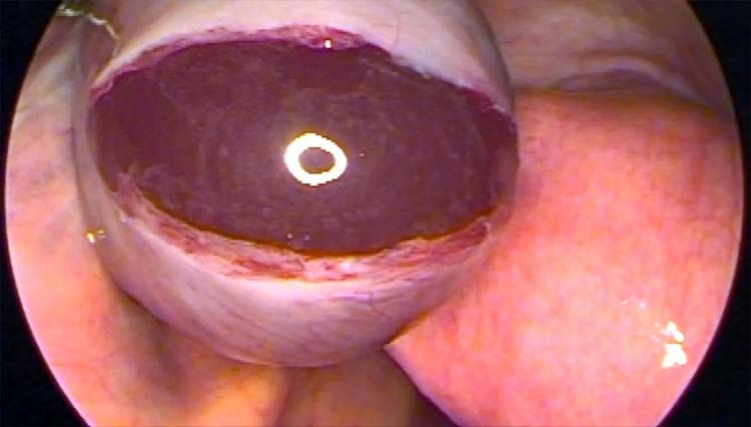
Laparoscopic image showing the ovarian stigma of the ruptured thick tunica albuginea in PCOS patients.

The patient was diagnosed with polycystic ovary syndrome (PCOS) based on Rotterdam criteria [1]. The ovulation was inducted by letrozole. Laparoscopy was indicated for tubal factor, and it was done in the period of ovulation. Half an hour before undergoing laparoscopy, an acute pain in the left iliac region was reported by the patient. During the laparoscopy, we noticed the ovarian stigma and the rupture of the thick tunica albuginea (Fig. 1, Supplementary Video S1).

PCOS is a common, chronic endocrine disorder in women of reproductive age. It is characterized by infertility, menstrual dysfunction and hyperandrogenism that may be associated with hirsutism, acne, seborrhea and obesity, and it can also lead to having depression symptoms [2]. Rotterdam criteria for the diagnosis of PCOS must meet two of three of the following: oligo/anovulation, hyperandrogenism (clinical or biochemical) and polycystic ovaries on ultrasound [1]. The pathological features of the ovaries of women with PCOS are described as having multiple follicular cysts and fibrotic thickening of the tunica albuginea and cortical stroma, explaining the acute ovulation pain in PCOS patients [3].

## Supplementary Material

Ovulation_in_PCOS_Final_(2)_rjab482
